# TNF-α activates RELA expression via TNFRSF1B to upregulate OPA1 expression and inhibit chondrogenic differentiation of human adipose stem cells

**DOI:** 10.1186/s13018-023-03846-x

**Published:** 2023-06-13

**Authors:** Jiajia Guo, Wang Ye, Xinglin Wu, Haifeng Huang, Bo Li, Zeyu Sun, Zhijing Ren, Zhen Yang

**Affiliations:** 1 Medical College of Guizhou University, Guiyang, 550025 Guizhou China; 2 Department of Orthopedics, Guizhou Provincial People’s Hospital, Guiyang, 550002 Guizhou China; 3 Department of Clinical Laboratory, Guizhou Provincial People’s Hospital, Guiyang, 550002 Guizhou China

**Keywords:** OPA1, Mitochondrial fusion, TNFRSF1B, RELA, Chondrogenic differentiation, Human adipose-derived stem cells

## Abstract

**Background:**

Tumor necrosis factor-alpha (TNF-α), one of the pro-inflammatory cytokines mediating the local inflammatory process in joints, inhibits cartilage formation and has a detrimental effect on stem cell-based cartilage regeneration for the treatment of osteoarthritis (OA). However, the mechanisms behind this inhibitory effect are still poorly understood. Mitochondrial morphological changes mediated by mitochondrial fusion and fission are highly plastic, are quite sensitive to environmental stimuli and play a crucial role in maintaining cell structure and function. In our study, chondrogenic differentiated human adipose stem cells (hADSCs) were exposed to TNF-α and the effect of TNF-α on the ability of hADSCs to chondrogenic differentiate and on mitochondrial fusion and fission was observed and analyzed. The aim was to investigate the role and mechanisms of mitochondrial fusion and fission regulation in the chondrogenic differentiation of hADSCs under normal conditions and under exposure to TNF-α.

**Methods:**

We used flow cytometry to identify hADSCs immunophenotypes CD29, CD44, CD34, CD45, and HLA-DR. Alcian blue staining and Sirius red staining were used to observe the formation of proteoglycans and collagen during the chondrogenic differentiation of hADSCs, respectively. The mRNA and protein expression levels of the cartilage formation marker SOX9, type II collagen (COL2A1), and Aggrecan were measured by real-time fluorescent quantitative PCR (RT-qPCR) and western blot, respectively. The fluorescent probes MitoTracker® Red CMXRos and JC-1 were used to visualize mitochondria morphology and detect mitochondrial membrane electricity (MMP). Affymetrix PrimeView™ chips were used for gene expression profiling.

**Results:**

The results showed that the chondrogenic differentiation of hADSCs was inhibited in the presence of TNF-α that optic atrophy 1 (OPA1) expression was significantly upregulated and mitochondria were prolonged and interconnected during this process. Gene microarray and RT-qPCR data showed that the presence of TNF-α led to increased expression of TNFα receptor 2 (TNFRSF1B) and RELA during chondrogenic differentiation of hADSCs.

**Conclusions:**

TNF-α inhibits chondrogenic differentiation of human adipose stem cells by activating RELA expression through TNFRSF1B upregulating OPA1 expression thereby increasing mitochondrial fusion.

## Background

Osteoarthritis (OA) is a progressive disease involving all joint structures, with a heterogeneous syndrome of different clinical phenotypes [1]. The clinical symptoms of OA are pain and functional impairment [2], and there is a lack of effective treatment for OA. The current treatment for OA aims to reduce the progression of the disease, treat the symptoms, reduce pain, and ensure as much mobility and function of the joint as possible in the late stages [3]. Cartilage tissue engineering is an emerging and effective approach to treating OA [4]. Human adipose stem cells (hADSCs) are a type of stem cell with multidirectional differentiation potential isolated from adipose tissue. They can be obtained from liposuction or lipectomy by-products, are less damaging to the donor [5, 6], and have better immunomodulatory ability, making them ideal seed cells for cartilage tissue engineering [7].

Tumor necrosis factor-alpha (TNF-α) is a pro-inflammatory factor that often mediates the local inflammatory process in joints. TNF-α has a significant inhibitory effect on articular cartilage formation. As cartilage destruction increases, TNF-α secretion in synovial tissue and joint fluid gradually increases [8]. In the pro-inflammatory cascade, TNF-α eventually activates the NF-κB/RELA transcription pathway, then secretes IL-6 and IL-8, accelerating the chondrocyte senescence and cartilage degeneration, leading to the aggravation of OA symptoms [9]. It was found that TNF-α inhibits the synthesis of SOX9, a key transcription factor required for cartilage differentiation [10], and suppresses the synthesis of cartilage matrix type II collagen (COL2A1) [11] and Aggrecan [12]. However, stem cell-based treatments for OA are often in the inflammatory environment in the presence of TNF-a, which can make the treatment much less effective or ineffective.

Mitochondria are highly dynamic organelles that play a crucial role in various biological processes in stem cells [13]. Mitochondrial fusion allows for exchanging material and connections within the mitochondria, providing sufficient energy, mitigating oxidative damage, and maintaining the mitochondrial membrane potential (MMP) [14]. Mitochondrial fission provides sufficient mitochondria to maintain cell polarity and eliminate damaged mitochondria [15]. Mitochondrial fusion and fission are regulated by a series of evolutionarily conserved proteins and kinetically related uridine diphosphate enzymes. Optic atrophy protein (OPA1), located in the inner mitochondrial membrane, primarily mediates the fusion of the inner mitochondrial membrane [16], and dynamin-related protein 1 (Drp1) mediates mitochondrial fission [17]. Mitochondria are the main physiological source of intracellular reactive oxygen species (ROS) [18]. ROS is essential for stem cell differentiation, and extended mitochondria in stem cells maintain low ROS levels and promote self-renewal. In contrast, the shift to a fragmented state of mitochondria leads to a moderate increase in ROS levels, which can inhibit self-renewal and differentiation gene expression [19]. This change in mitochondrial morphology, mediated by mitochondrial fusion and fission, is highly plastic and sensitive to environmental stimuli. To meet the specific functional needs of cells, mitochondrial networks are different among different types of stem cells, different pluripotent states, and specific commitment destinies [20].

This study used TNF-α to induce cells to form an OA cell model at the cellular level to investigate the specific effects of TNF-α on mitochondrial fusion/fission during the chondrogenic differentiation of hADSCs and its mechanism, providing a new therapeutic strategy for the treatment of OA.

## Methods

### hADSCs culture and immunophenotyping

We have obtained hADSCs from the by-products of liposuction in healthy humans [21]. Second-generation cells were removed from the liquid nitrogen and rapidly resuscitated and cultured in an H-DMEM medium (Pricella, China) containing 10% FBS (Gibco, USA) and 1% penicillin/streptomycin (Sigma, USA). hADSCs were enzymatically separated using 0.25% trypsin–EDTA solution when their proliferation was above 80%, and they were then passed in a 1:3 ratio. Fourth-generation hADSCs are used for experiments. hADSCs were collected and incubated with direct-coupled antibodies CD29 (1:100; Cat. No. 11-0299-42; Thermo, USA), CD44 (1:100; cat. no. 11-0441-85; Thermo, USA), CD34 (1:100; Cat. No. CD34-581-01; Thermo, USA), CD45 (1:100; Cat. No. 12-9459-42; Thermo, USA), and HLA-DR (1:100; Cat. No. 12-9956-42; Thermo, USA) on ice for 1 h. After incubation, the cells were analyzed by NovoCyte 2060R flow cytometer (ACEA Biosciences, USA).

### Chondrogenic, adipogenic and osteogenic differentiation of hADSCs

The multidirectional differentiation of hADSCs was induced by microsphere culture. First, hADSCs were collected in 15 ml centrifuge tubes, and then induced differentiation was carried out with adipogenic differentiation, chondrogenic differentiation, and osteogenic differentiation medium, respectively. The configuration of differentiation medium is  shown in Table 1, and the final differentiation was verified by staining after 14, 21, and 28 days. At the end of the induction of differentiation, the cell microspheres were fixed in 4% paraformaldehyde (Servicebio, China) for 1 h. Adipogenic differentiated cells were prepared in 10 μm sections through a frozen section. Suitable sections were selected to be stained first with Oil Red O dye (Servicebio, China) and then re-stained with hematoxylin dye (Servicebio, China). Chondrogenic and osteogenic differentiated cells are prepared in 5 μm thick sections by paraffin sectioning. The sections are dewaxed and placed in water. Suitable sections were selected for staining in Alcian Blue, Sirius Red, and Alizarin Red dyes (Servicebio, China). After the staining was completed, observation and photography were carried out using a microscope. Image J software (National Institutes of Health, USA) statistically analysed the percentage of the positive area of staining results.

**Table 1:** Induced differentiation medium preparation

Induced differentiation medium	Reagent preparation concentration
Adipogenic differentiation medium	89%H-DMEM + 1%(Streptomycin and Penicillin) + 10% FBS + 10 μg/ml Insulin + 1 μM Dexamethasone (Solarbio, China) + 200 μM Indomethacin + 0.5 mM 3-Isobutyl-1-methylxanthine(IBMX)
Chondrogenic differentiation medium	89%H-DMEM + 1%(Streptomycin and Penicillin) + 10% FBS + 10 ng/ml TGF-β1(MCE, USA) + 50 μg/ml Vc(Solarbio, China) + 1 μM Dexamethasone + 6.25 μg/ml Insulin(MCE, USA)
Osteogenic differentiation medium	89%H-DMEM + 1%(Streptomycin and Penicillin) + 10%FBS + 1 μM Dexamethasone + 10 mM beta-glycerophosphate disodium + 50 μg/ml Vc

### Experimental grouping and stimulation with cytokines

The hADSCs induced to differentiate were divided into two groups, the Chondro group and the Chondro-TNF-α group. In Chondro group, chondrogenic differentiation medium was used to induce differentiation. Chondro-TNF-α group stimulates cells and induces differentiation by adding recombinant TNF-α (MCE, USA) of 10 ng/ml to chondrogenic differentiation medium.

### Real‑time quantified PCR

Total RNA was extracted from the cells using the TRIzol (Invitrogen, USA) reagent, and the concentration and purity of the extracted RNA were assessed using a micro-nucleic acid protein concentration meter (Bio-Sun, China) to measure absorbance values at 260 and 280 nm. PrimeScript RT (Invitrogen, USA) reagent was used for reverse transcription synthesis of cDNA. SYBR Premix Ex Taq kits (Invitrogen, USA) are used for RT-qPCR experiments. Glyceraldehyde-3-phosphate dehydrogenase (GADPH) was used as the housekeeping gene. The 20 µl RT-qPCR system mixture includes SYBR Green Premix (10 µl), cDNA (2 µl), forward primer (10 µm; 0.8 µl), reverse primer (10 µm; 0.8 µl), Dey II (0.4 µl), enzyme-free water (6 µl). The reaction procedure is 30 s at 95 °C; 5 s at 95 °C and 30 s at 60 °C and for 40 cycles; 15 s at 95 °C, 30 s at 60 °C, and 15 s at 90 °C. For the primer sequences used, see Table 2. The results were analyzed using the 2^−ΔΔCt^ method.

**Table 2:** Primers used in this study

Primer	Sequence (5′–3′)	
SOX9	Forward: CAGCGAACGCACATCAAGACG	Reverse: TGTAGGTGAAGGTGGAGTAGAGGC
COL2A1	Forward: CGTGGACGATCAGGCGAAAC	Reverse: AAGCCAGCAAAGGCGGACAT
Aggrecan	Forward: ACTGCCTTCGCTGAGGTTGA	Reverse: CACTGCTCATAGCCTGCTTCGT
GAPDH	Forward: AAGGCTGTGGGCAAGGTCAT	Reverse: GGAGGAGTGGGTGTCGCTGT
DNM1L	Forward: CTGAGGCTGATGGCAAGTT	Forward: GAAGAGCAGCGTGGGGACT
OPA1	Forward: CAGACTGGAAAAAGAGGTG	Reverse: CGACAAAGGTTACAATGGT
RELA	Forward: GAAGAGCAGCGTGGGGACT	Reverse: TGCACATCAGCTTGCGAAA
TNFRSF1B	Forward: TGGACTGATTGTGGGTGTG	Reverse: CTGGTGCCTGTGGCTGGTT

### Western blot analysis

High-performance RIPA lysate (Solarbio, China) was used for protein isolation and extraction. The BCA protein concentration assay kit (Solarbio, China) was used to determine the total protein concentration of the cells. Proteins were separated by SDS-PAGE with a protein loading volume of 30 μg per well. Isolated proteins were transferred onto PVDF membranes (Millipore, USA) by electrotransfer and then mixed with proportionally diluted primary antibodies SOX9 (1:2000; Cat. No. 67439-1-Ig; Proteintech, China), COL2A1 (1:2000; Cat. No. 28459-1-AP; Proteintech, China), Aggrecan (1:500; Cat. No. 13880-1-AP; Proteintech, China), and GAPDH (1:10,000; Cat. No. 60004-1-Ig; Proteintech, China) for 2 h at room temperature in a shaker incubation. Then incubate for one hour in the corresponding mouse (1:10,000; Cat. No. SA00001-1; Proteintech, China)/rabbit (1:10,000; Cat. No. SA00001-2; Proteintech, China) secondary antibody solution on a shaker. After incubation development exposures and photographs were taken and the results were analyzed using Image J software (National Institutes of Health, USA).

### Active mitochondria staining

HADSCs were inoculated in six-well plates, and when the proliferation of hADSCs was observed to be confluent to about 40%, the medium was changed to the corresponding induction medium. At the end of induction differentiation, MitoTracker® Red CMXRos (200 nM, 1 ml; Solarbio, China) staining solution was added and incubated in an incubator at 37 °C 20 min. After the incubation, they were quickly observed under a fluorescent microscope and photographed.

### ROS detection by flow cytometry

Intracellular ROS levels were detected using a reactive oxygen species kit (Beyotime, China). HADSCs were inoculated in six-well plates and observed to be at approximately 70% cell proliferation confluence when replaced with the appropriate induction differentiation medium. After induction of differentiation, the cells were resuspended in DCFH-DA solution (10 μM/L, 1 ml) diluted 1:1000 with serum-free medium and incubated for 30 min at 37 °C in an incubator, and assayed and analyzed by flow cytometry at the end of incubation.

### MMP assay analysis

HADSCs were inoculated in six-well plates and cell proliferation was observed at approximately 40% confluence, and the medium was replaced with the appropriate differentiation medium. At the end of differentiation, 1 ml of JC-1 staining (Beyotime, China) working solution was added, and the cells were incubated for 20 min at 37 °C in a cell incubator. After incubation, the cells were observed under a fluorescent microscope and photographed.

### Gene expression profiling

Total RNA from the cells was extracted, and the concentration and purity of the RNA were checked by NanoDrop microspectrophotometer (Thermo, USA), followed by agar gel electrophoresis to determine RNA integrity and the absence of DNA contamination. After library construction and quality control, the fragmented cRNAs were added to Affymetrix PrimeView™ chips (Cnkingbio, China), hybridized and washed, and then scanned using a GeneChip 300 7G scanner (Thermo, USA). Agilent Feature Extraction software (Agilent Technologies, USA) was used to process the gene chip scans and obtain the raw data. Gene-Spring software (Agilent Technologies, USA) was used to normalize the analysis and export the data in Excel format. The screening criteria for differentially expressed genes were fold change (FC) ≥ 2.0 and *P* < 0.05.

### Statistical analysis

The experimental data were expressed as mean ± standard deviation and processed using GraphPad Prism 8 software (GraphPad Software, USA). Comparisons between groups were made using independent samples t test and one-way ANOVA. Comparisons between groups were statistically significant at *P* < 0.05.

## Results

### Characterization of hADSCs

**Fig. 1:**
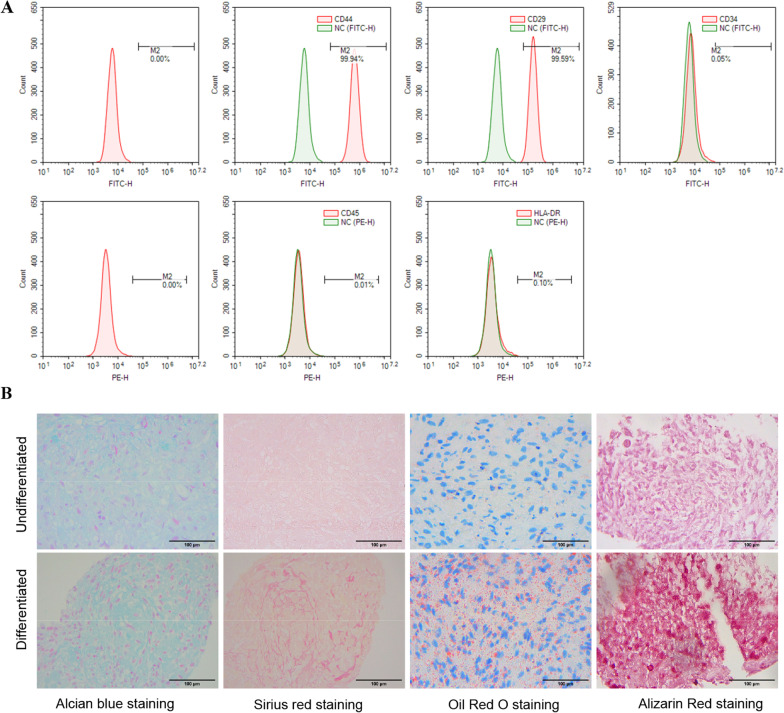
Immunophenotypic and multidirectional differentiation potential of hADSCs. **A** Flow cytometric analysis of surface marker expression on MSCs. They were positive for CD44 and CD29, but negative for CD45, CD34, and HLA-DR. **B** The hADSCs not induced to differentiate and those induced to differentiate were stained with Arsenic Blue, Sirius Scarlet, Oil Red O, and Alizarin Red, respectively. All the uninduced differentiated ones showed negative results, and all the induced differentiated ones showed positive results. Scale bars = 100 μm

The immunophenotype of hADSCs was detected by flow cytometry. The results showed that more than 99% of the cells expressed CD29 and CD44 and less than 0.1% expressed CD34, CD45, and HLA-DR (Fig. 1A). To verify that the cells used in the experiments have a multidirectional differentiation potential, cells were induced to differentiate into chondrocytes, adipocytes, and osteocytes, respectively. Cell differentiation was observed by specific staining. Alcian blue and Sirius red staining were used to show the chondrogenic tissue components proteoglycans and collagen, respectively, to determine cellular differentiation into chondrocytes. Oil Red O and Alizarin Red staining are primarily used to identify lipid droplets and mineralized stroma to determine cellular differentiation into adipocytes and osteoblasts, respectively. All staining showed positive results (Fig. 1B), indicating that the hADSCs used in the experiments had multidirectional differentiation potential.

### TNF-α inhibits chondrogenic differentiation of hADSCs

**Fig. 2:**
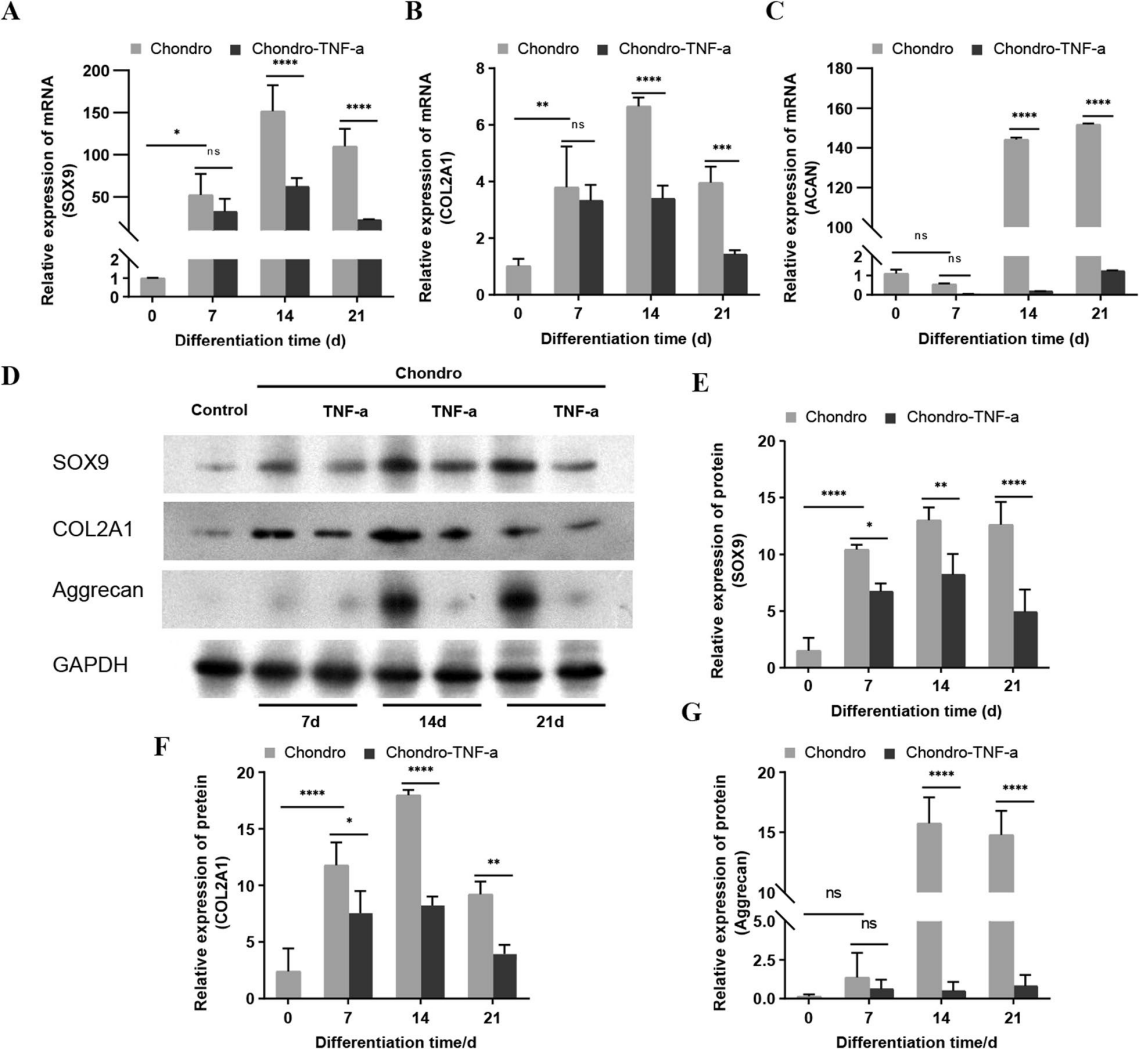
Changes in mRNA and protein expression levels of SOX9, COL2A1, and Aggrecan, markers of chondrogenesis, during chondrogenic differentiation of hADSCs. **A**–**C** The mRNA expression of SOX9, COL2A1, and Aggrecan was measured by RT-qPCR after 0, 7, 14, and 21 days of chondrogenic differentiation in hADSCs. **D** Protein expression of SOX9, COL2A1, and Aggrecan was measured by western blot after 0, 7, 14, and 21 days of chondrogenic differentiation in hADSCs. **E**–**G** Calculate the integral gray value of protein bands for quantitative analysis. Mean ± SD of three independent experiments; ****P < 0.0001, ***P < 0.001, **P < 0.01, *P < 0.05, ns = not significant; d: day

Studies have shown that the effect of TNF-α on the chondrogenic differentiation of bone marrow mesenchymal stem cells (BMSCs) is dose-dependent, and 10 ng/ml of TNF-α reagent to the chondrogenic differentiation medium has a significant inhibitory effect on the chondrogenic differentiation of BMSCs [12]. The hADSCs were divided into two groups, the Chondro group cultured in a chondrogenic differentiation medium and the Chondro-TNF-α group cultured in a chondrogenic differentiation medium with 10 ng/ml TNF-α reagent. Both groups were induced into chondrogenic differentiation for 7, 14, and 21 days, respectively. The mRNA and protein expression of SOX9, COL2A1, and Aggrecan, markers of chondrogenesis was examined by RT-qPCR and western blot, in both groups after induction of chondrogenic differentiation. The results showed that the mRNA and protein expression levels of SOX9, COL2A1, and Aggrecan were significantly upregulated in both Chondro and Chondro-TNF-α groups compared to undifferentiated (day 0 of differentiation) cells, except for Aggrecan in the Chondro-TNF-α group. The mRNA (Fig. 2A–C) and protein (Fig. 2D–G) expression levels of SOX9, COL2A1, and Aggrecan were significantly lower in the Chondro-TNF-α group than in the Chondro group at 14 and 21 days of chondrogenic differentiation.

**Fig. 3:**
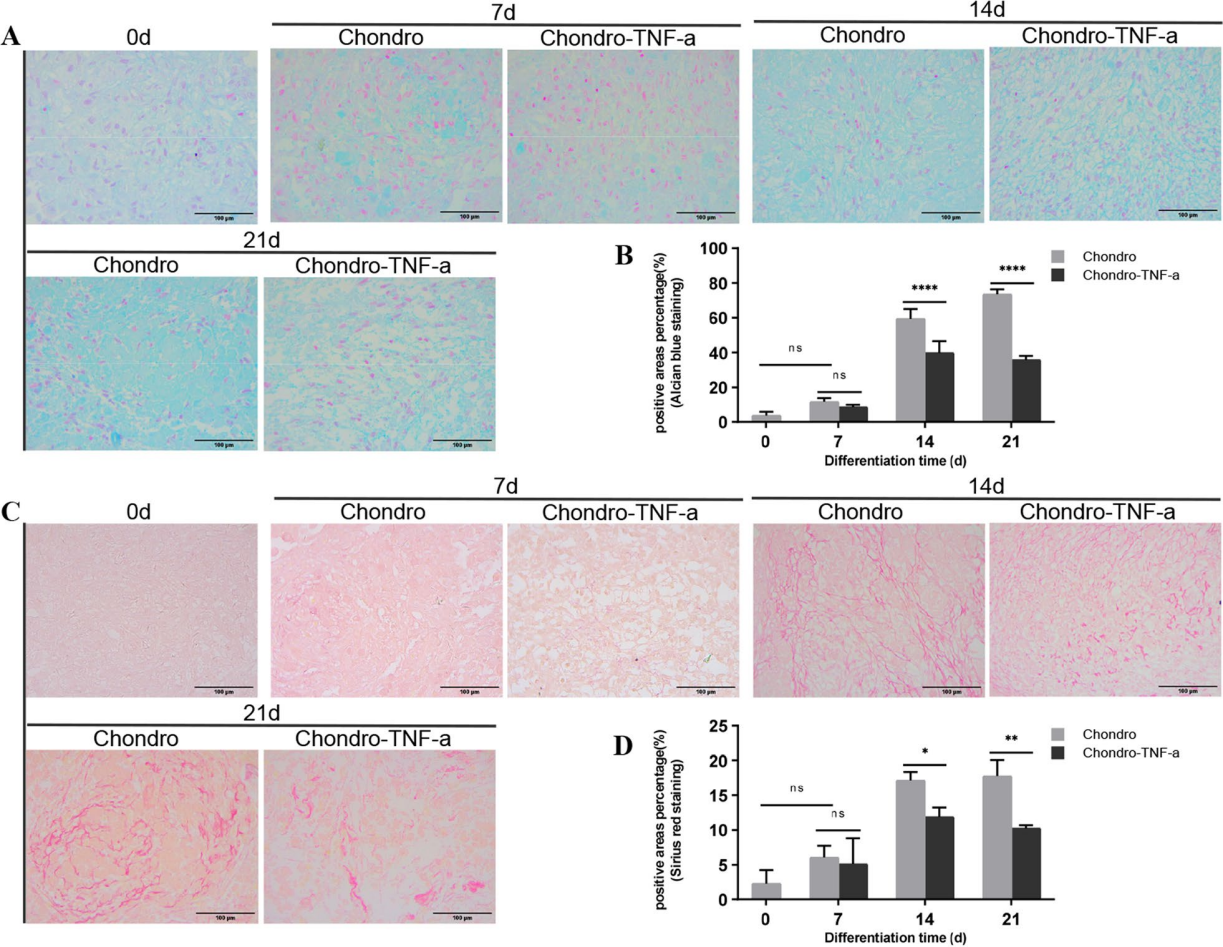
Formation of proteoglycans and collagen during cartilage differentiation in hADSCs. **A** Protein polyaminoglycan formation was observed with Alcian blue staining. **B** Histogram of quantitative analysis of Alcian blue staining. **C** Collagen formation was observed with Sirius red staining. **D** Histogram of quantitative analysis of Sirius red staining. Mean ± SD of three independent experiments; Scale bars = 100 μm; ****P < 0.0001, **P < 0.01, *P < 0.05; ns = not significant; d: day

Histological staining of the Chondro group and Chondro-TNF-α group at different differentiation times was observed using Alcian blue staining and Sirius red staining. At 14 and 21 days of chondrogenic differentiation in hADSCs, the percentage of positive areas for Alcian blue staining (Fig. 3A, B) and Sirius red staining (Fig. 3C, D) were significantly lower in the Chondro-TNF-α group than in the Chondro group. These results suggest that the presence of TNF-α during the chondrogenic differentiation of hADSCs reduces the differentiation potential of hADSCs.

### TNF-α promotes mitochondrial fusion in the prophase of chondrogenic differentiation of hADSCs

**Fig. 4:**
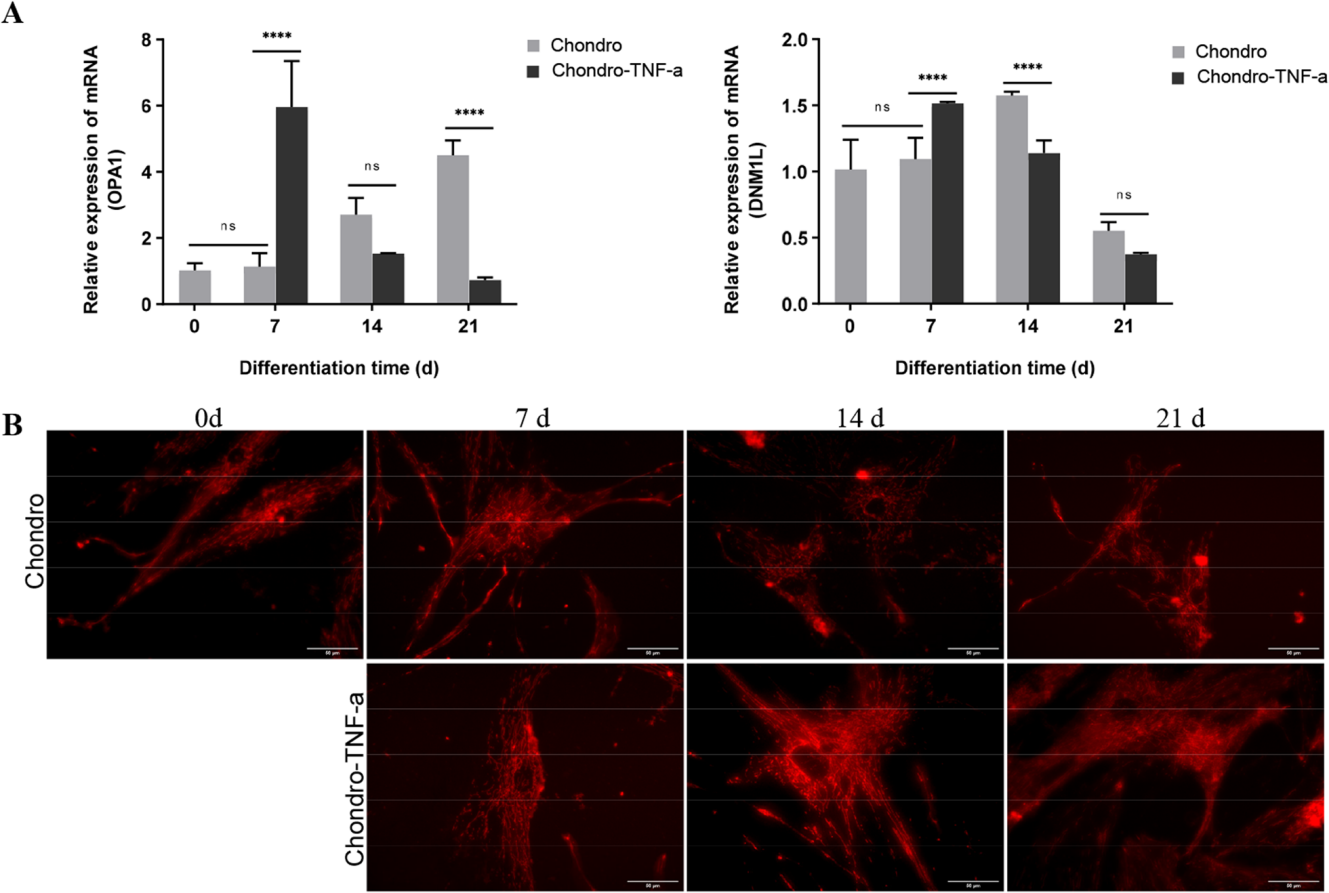
Changes in the mitochondrial fusion and fission level during chondrogenic differentiation of hADSCs. **A** Expression of mRNA of mitochondrial fusion gene OPA1 and mitochondrial fission gene DNM1L during chondrogenic differentiation of hADSCs was detected by RT-qPCR; **B** mitochondria of hADSCs were labeled with a fluorescent dye (in red) to observe the changes in mitochondrial morphology during chondrogenic differentiation of hADSCs. Mean ± SD of three independent experiments; Scale bars = 50 μm; ****P < 0.000, ns = not significant; d: day

The changes in mRNA expression levels of OPA1 and Drp1 during chondrogenic differentiation of hADSCs were detected by RT-qPCR. Unlike mesenchymal stem cells (MSCs), which exhibit a tendency for mitochondrial fusion and increased OPA1 expression during adipogenic and osteogenic differentiation, MSCs exhibit a tendency for mitochondrial fission and increased Drp1 expression during chondrogenic differentiation [22]. The mRNA expression levels of OPA1 in the Chondro group were significantly upregulated at day 14 of differentiation and continued to be upregulated until day 21 of differentiation, at which point OPA1 expression was significantly higher in the Chondro group than in the Chondro-TNF-α group, and the mRNA expression levels of Drp1 were highest at day 14 of differentiation and significantly higher than in the Chondro-TNF-α group; the mRNA expression levels of OPA1 and Drp1 in the Chondro-TNF-α group were significantly upregulated at 7 days of differentiation, much higher than at other differentiation time points. The upregulation of OPA1 was much higher than that of Drp1, which was significantly down-regulated at 14 days of differentiation and continued to be down-regulated until 21 days of differentiation when both expression levels were lower than in undifferentiated (day 0 of differentiation) cells (Fig. 4A).

To observe changes in mitochondrial morphology during chondrogenic differentiation in both groups of hADSCs, we labeled the mitochondria with a red fluorescent probe. Using a fluorescence microscope, it was possible to observe that the Chondro group at 14 days of differentiation hADSCs mitochondria underwent significant fragmentation and were short and granular; on the 7th day of chondrogenic differentiation of hADSCs in Chondro-TNF-a group, the appearance of mitochondria was elongated, slender, and connected into a network (Fig. 4B).

### The presence of TNF-α reduces ROS production during the chondrogenic differentiation of hADSCs and somewhat attenuates the decrease in mitochondrial membrane potential (MMP)

**Fig. 5:**
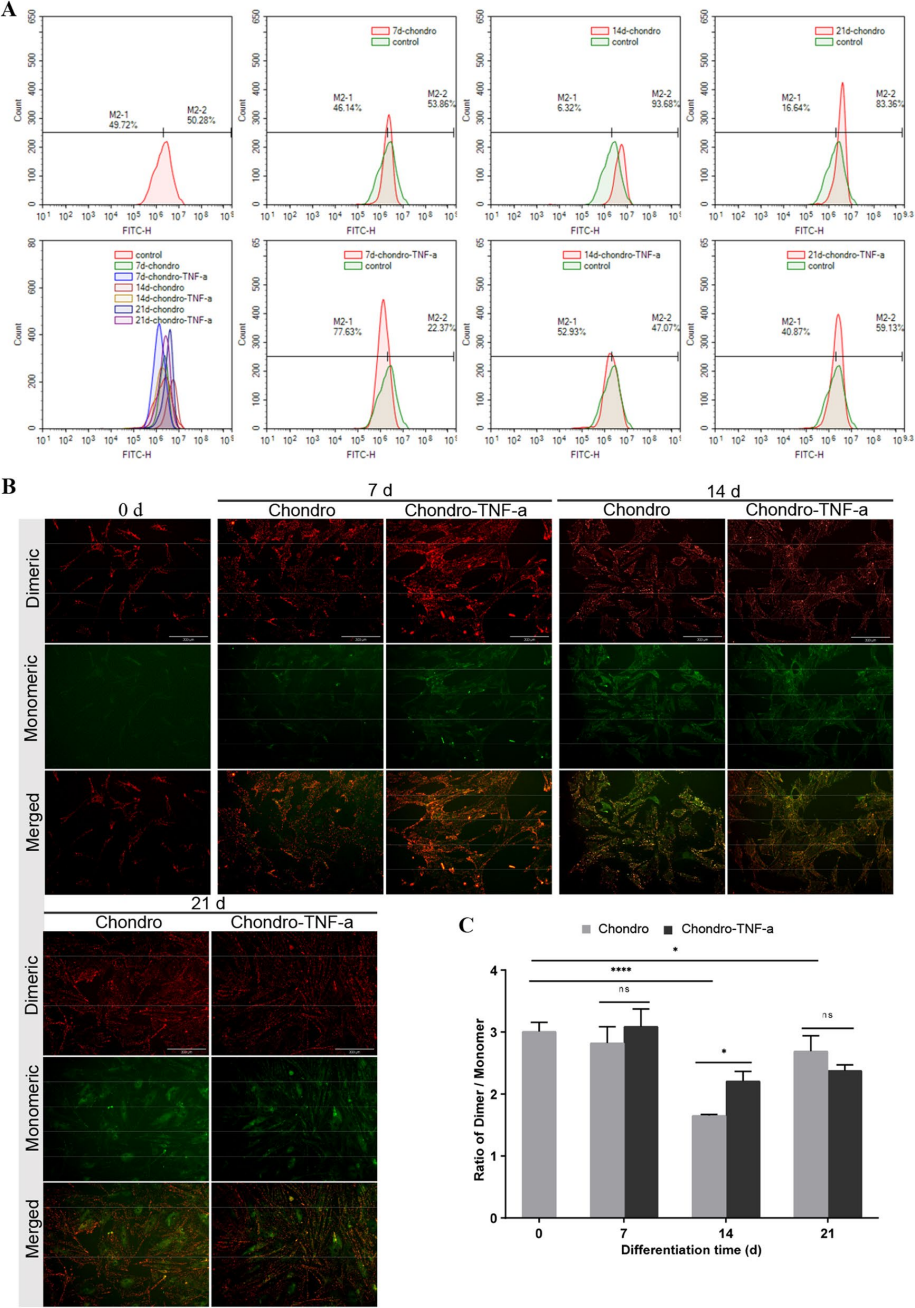
Changes in ROS levels and MMP during chondrogenic differentiation of hADSCs. **A** DCFH-DA fluorescent probes were mounted within entry hADSCs for ROS detection by flow cytometry. **B** JC-1 fluorescent probe detects MMP with a red fluorescence from the dimer and a green fluorescence from the monomer. **C** The ratio of dimer to monomer, with a higher ratio indicating higher MMP and vice versa. Mean ± SD of three independent experiments; Scale bars = 50 μm; ****P < 0.0001, *P < 0.05, ns = not significant; d: day

Intracellular ROS and MMP are closely related to mitochondrial function. Flow cytometry was used to detect changes in ROS levels during the chondrogenic differentiation of hADSCs. The results showed that the Chondro group showed the most significant increase in ROS levels at 14 days of differentiation, which was higher than that of the other differentiation time points and the Chondro-TNF-α group. The Chondro-TNF-α group showed significant downregulation of ROS levels at 7 days of differentiation and were lower than the control (0 days of differentiation) group (Fig. 5A). As differentiation continued, ROS levels increased in the Chondro-TNF-α group. Still, they were consistently lower than in the Chondro group at the same differentiation time point (Fig. 5A).

MMP is required for mitochondrial inner membrane fusion [23], and paired fission is activated when MMP is eliminated [24]. Changes in ROS have a regulatory effect on MMP, with an increase in ROS causing a decrease in MMP [25]. We use the JC-1 fluorescent probe to detect MMP. The ratio of the dimer (red fluorescence) to monomer (green fluorescence) can be used to determine the level of MMP. The results showed that the Chondro group had the lowest MMP at 14 days of differentiation, which was lower than that of the other differentiation time points and the Chondro-TNF-α group (Fig. 5B, C). The Chondro-TNF-α group also showed a significant decrease in MMP at 14 days of differentiation, but not to the same extent as the Chondro group, which continued to differentiate, with no significant change in MMP in the Chondro-TNF-α group (Fig. 5B, C). These results suggest that the process of chondrogenic differentiation of hADSCs involves a moderate increase in ROS and a moderate decrease in MMP. However, the presence of TNF-α reduced the production of ROS during the chondrogenic differentiation of hADSCs and somewhat attenuated the decrease in MMP.

### TNF-α upregulates OPA1 expression by activating RELA expression through TNFRSF1B

**Fig. 6:**
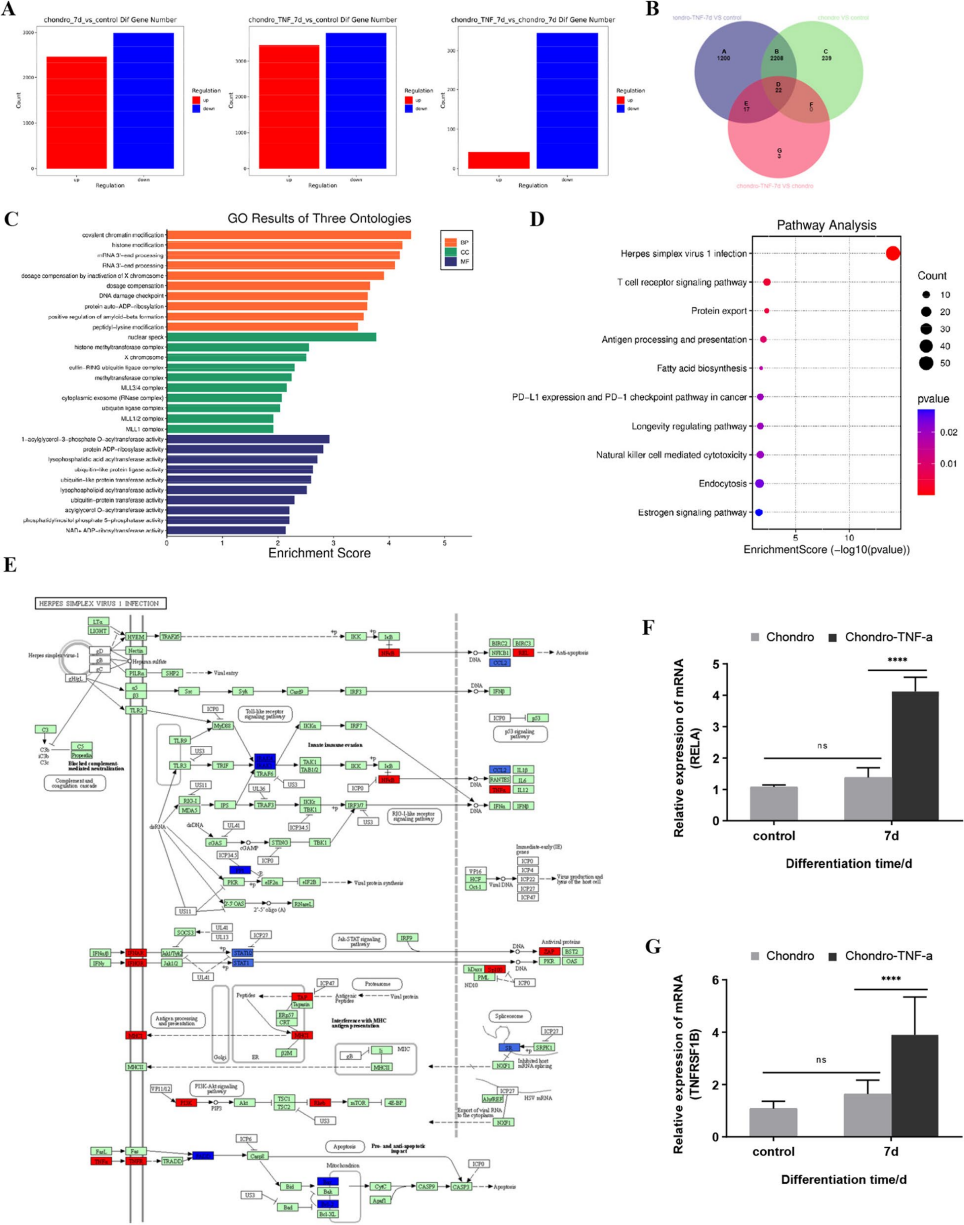
Screening of DEGs, GO, and KEGG enrichment analysis. **A** Two-by-two comparison of gene expression profile assay data for the number of up- and down-regulated DEGs. **B** A Wayne diagram showing the screened 1242 (A + D + E + G) DEGs. **C** The GO functional enrichment analysis of the screened DEGs shows the top ten enrichment rankings, respectively. Orange indicates biological processes (BP), green indicates cellular components (CC), and dark blue indicates molecular functions (MF). **D** KEGG enrichment analysis of screened DEGs showing the top ten KEGG pathways in terms of enrichment. The x-axis shows the enrichment score, the y-axis shows pathway names, and the point size represents the number of genes enriched in a particular pathway. **E** KEGG pathway enrichment analysis and “Herpes simplex virus 1 infection” pathway map. Red indicates upregulated genes, dark blue indicates down-regulated genes, green indicates the presence of the gene in the species, and white indicates the absence of the gene in the species. **F**–**G** Expression levels of TNFRSF1B and RELA in the “Herpes simplex virus 1 infection” signaling pathway were measured by RT-qPCR. ****P < 0.0001, ns = not significant; d: day

The results of the previous experiments were summarized by observing that the expression levels of OPA1, DNM1L, mitochondrial morphology, and ROS levels were significantly different in the Chondro-TNF-α group compared to the Chondro group at 7 days of chondrogenic differentiation. Therefore, we prepared hADSCs from the Chondro group and Chondro-TNF-α group into cartilage differentiation at 7 days and hADSCs from the control group (differentiation 0 d) for gene expression profiling. The results showed that the Chondro-TNF-α group involved up- and down-regulated differentially expressed genes (DEGs) significantly more than the Chondro group (Fig. 6A). We focused our study’s interest on upregulated DEGs. We screened 1242 DEGs for upregulation in the Chondro-TNF-α group versus control group by subtracting the same genes from the upregulated DEGs in the Chondro group versus control group and adding the upregulated DEGs in the Chondro-TNF-α group versus Chondro group for in-depth analysis (Fig. 6B).

Relevant Gene Ontology (GO) and Kyoto Encyclopedia of Genes and Genomes (KEGG) signaling pathway enrichment analyses were performed on the screened DEGs. GO analysis showed that DEGs are mainly involved in biological processes such as “covalent chromatin modification,” “histone modification,” and “mRNA 3'-end processing” (Fig. 6C). KEGG analysis showed that the signaling pathways with high enrichment were mainly “Herpes simplex virus 1 infection,” “T cell receptor signaling pathway,” and “Protein export” (Fig. 6D). It was shown that TNFRSF1B stimulation synergistically increases OPA1 transcription through STAT3 and RELA binding to adjacent regions of the OPA1 promoter, which in turn upregulates OPA1 expression and increases mitochondrial fusion [26]. We measured the expression levels of TNFRSF1B and RELA in the signaling pathway of “Herpes simplex virus 1 infection” by RT-qPCR (Fig. 6E) and found that the results were consistent with the gene microarray data (Fig. 6F) and suggest that TNF-α has a regulatory effect on mitochondrial function during chondrogenic differentiation of hADSCs and that this effect is mediated at least in part by the upregulation of OPA1 expression through TNFRSF1B–RELA signaling.

## Discussion

There is already a lot of research data confirming that TNF-α inhibits the chondrogenic differentiation of stem cells [10, 12, 27]. However, the mechanisms behind these inhibitory effects are still poorly understood. In this study, we found for the first time that the presence of TNF-α upregulated the expression of OPA1 during chondrogenic differentiation of hADSCs, and the morphology of mitochondria tended to lengthen and connect to form a network. Microarray data show that the presence of TNF-α can significantly upregulate the expression of TNFRSF1B and RELA during the chondrogenic differentiation of hADSCs. In summary, we found that TNF-α can upregulate the expression of OPA1 by activating RELA expression by TNFRSF1B and increase mitochondrial fusion to some extent.

In most cases, the use of stem cells for cartilage regeneration treatment OA is in an inflammatory environment. However, inflammatory environments inhibit cartilage formation and accelerate cartilage destruction [12]. This would substantially reduce the potential for chondrogenic differentiation of stem cells and the therapeutic effect. There is growing evidence that TNF-α and Interleukin-1β (IL-1β) are important pro-inflammatory mediators in OA. These cytokines play a crucial role not only in the regulation of subchondral bone resorption but also in the shift from endochondral homeostasis to catabolism [27]. As shown in our data, TNF-α significantly reduced cartilage-specific matrix synthesis and down-regulated the expression of chondrogenic marker genes. Data from several studies suggest that TNF-α significantly downregulates gene expression of SOX9, a transcription factor essential in the early stages of chondrogenesis and responsible for the subsequent inhibition of chondrocyte maturation [10, 27]. TNF-α selectively inhibits collagen production, Aggrecan synthesis, and Aggrecan degradation, leading to chondrocyte apoptosis and cartilage degeneration [28]. Silencing TNF-α in vivo can significantly promote the differentiation of BMSCs into chondrocytes and then promote cartilage regeneration in vivo [29]. Our study confirmed that TNF-α inhibits cartilage formation and significantly reduces the chondrogenic differentiation ability of hADSCs (Figs. 2, 3).

Altering mitochondrial dynamics is both a consequence and a cause of stem cell differentiation. The morphology of mitochondria and their regulatory processes of fusion and fission are regulated during the pre-differentiation phase of stem cells, leading to altered bioenergetic distribution during differentiation. The remodeling of the mitochondrial network is essential to the differentiation program, as these cells show a delayed or complete loss of differentiation when fusion or fission is abolished. During chondrogenesis, mitochondria are fragmented, Drp1 expression is increased, and the knockdown of Drp1 leads to a reduction in cell differentiation capacity [22]. During our experiment, we also observed that the expression of Drp1 increased and the mitochondria tended to be fragmented during cartilage formation. Our experimental data strongly support the view that mitochondrial fission is involved in cartilage formation.

Mitochondrial fusion and fission coordination key-cytoprotective mechanisms for cell renewal and dynamic homeostasis under different external stresses [20]. Mitochondrial fusion may compensate for weak external stresses to promote better survival. Preliminary indications are that in TNF-α, mitochondria tend to lengthen to resist external stresses to promote better survival. In many cases, mitochondrial ground lengthening facilitates stress resistance [30]. TNF-α stimulation can activate NF-κB/RELA transcription pathway, and RELA plays an important role in determining the fate of MSCs. The activation of RELA promotes the proliferation of MSCs but inhibits its osteogenic and chondrogenic differentiation [31]. The cardioprotective effect of low concentration of TNF-α is attributed to the activation of TNFRSF1B. The activation of TNFRSF1B protects cardiomyocytes from stress by upregulating the expression of OPA1. This process is mediated by p300-mediated STAT3 acetylation and STAT3/RELA interaction to improve and maintain the morphology and function of mitochondria [26]. Our data show that in response to the pressure of TNF-α stimulation, RELA was selected to be activated and hADSCs chondrogenic differentiation was inhibited. And mitochondria tend to lengthen and increase the expression of OPA1, so the regulation of mitochondrial fusion and fission is more conducive to cell survival.

## Conclusions

**Fig. 7:**
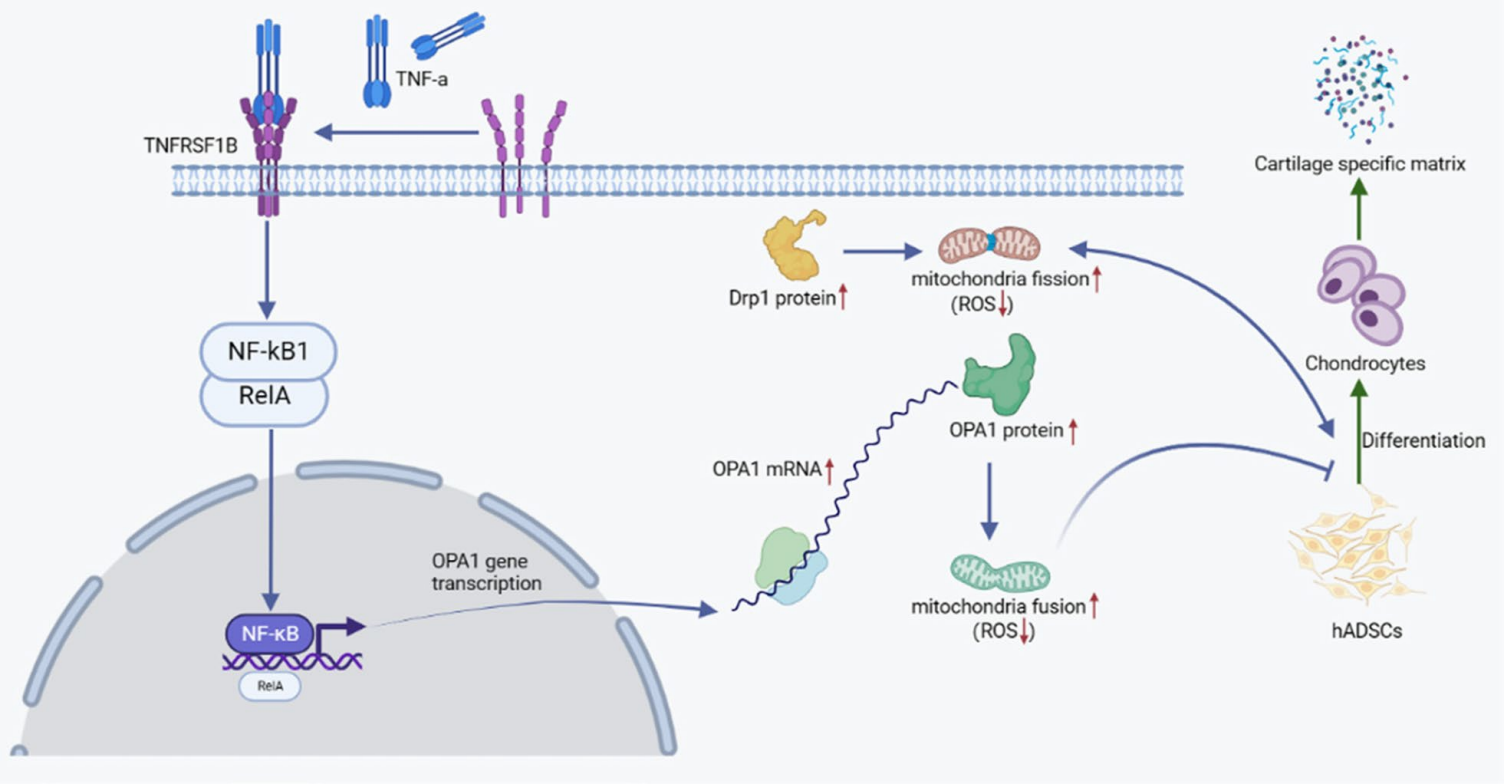
Schematic diagram: TNF-α upregulates OPA1 expression by activating RELA expression through TNFRSF1B, increasing mitochondrial fusion and inhibiting cartilage differentiation in hADSCs

In conclusion, we found that the mitochondria of hADSCs tended to fission and the expression of Drp1 increased during chondrogenic differentiation. However, TNF-α not only changes the chondrogenic differentiation ability of hADSCs, but also regulates the mitochondrial function in the process of chondrogenic differentiation of hADSCs, and this effect is mediated at least in part by the upregulation of OPA1 expression through TNFRSF1B–RELA signaling (Fig. 7). Therefore, when using hADSCs for cartilage regeneration in the treatment of OA, it can be used as a therapeutic or adjuvant target by downregulating the expression of TNFRSF1B to help increase mitochondrial fission and Drp1 expression required for cell differentiation.

## Data Availability

You may access the datasets applied in this study from the corresponding authors upon reasonable request.

## Abbreviations

TNF-α: Tumor necrosis factor-α
hADSCs: Human adipose-derived stem cells
OA: Osteoarthritis
COL2A1: Type II collagen
OPA1: Optic atrophy 1
TNFRSF1B: TNFα receptor 2
MMP: Mitochondrial membrane potential
Drp1: Dynamin-related protein 1
ROS: Oxygen species
GO: Gene Ontology
KEGG: Kyoto Encyclopedia of Genes and Genomics
